# The Capsaicin 8% Patch for Neuropathic Pain in Clinical Practice: A Retrospective Analysis

**DOI:** 10.1111/pme.12143

**Published:** 2013-05-24

**Authors:** Till Wagner, Chris Poole, Andrea Roth-Daniek

**Affiliations:** * Medizinisches Zentrum Städteregion AachenWürselen, Germany; † Pharmatelligence, Cardiff MediCentreCardiff, UK

**Keywords:** Capsaicin, Neuropathy, Chronic Pain, Polyneuropathy, Post-Herpetic Neuralgia, Pain Management

## Abstract

**Objective:**

To investigate the response of patients with peripheral neuropathic pain (PNP) to capsaicin 8% patch treatment in a clinical setting.

**Design:**

Retrospective analysis.

**Setting:**

The Clinic for Pain Therapy and Palliative Medicine at the Medical Centre for the region of Aachen, Germany.

**Subjects:**

Patients diagnosed with PNP who attended the clinic for capsaicin 8% patch treatment between January 13, 2010 and February 7, 2011.

**Outcome Measures:**

Pain intensity was assessed using the Numeric Pain Rating Scale (NPRS) at baseline and following each capsaicin 8% patch treatment. Changes in prescribed concomitant neuropathic pain (NP) medications and response duration were recorded.

**Results:**

Overall, 68 patients with PNP conditions, including facial neuropathy (severe trigeminal neuralgia in V2), polyneuropathy, post-herpetic neuralgia, and mononeuropathies, received 96 treatments with the capsaicin 8% patch. The 53 patients with a follow-up of ≥8 weeks demonstrated a 48.4% mean reduction in NPRS score from baseline to Weeks 1–8. Among the 37 responders (those exhibiting ≥30% reduction in NPRS score from baseline to Weeks 1–8), the median time to re-treatment was 125 days. Following treatment, there was a significant (*P* < 0.001) 54% reduction in the mean number of prescribed concomitant NP medications taken by patients.

**Conclusions:**

This analysis demonstrates that in clinical practice, the capsaicin 8% patch provides rapid and sustained pain reductions in patients with a variety of PNP conditions and a significant reduction in prescribed concomitant NP medications. The capsaicin 8% patch can be a valuable addition to the NP treatment armory for certain patients.

## Introduction

Neuropathic pain (NP) is pain that arises as a result of disease or damage to the somatosensory system related to a variety of underlying causes, including infections and physical injury 1,2. NP conditions include post-herpetic neuralgia (PHN), HIV-associated neuropathy (HIV-AN), painful diabetic neuropathy, radiculopathy, failed back surgery syndrome (FBSS), facial neuropathy (including trigeminal neuralgia), polyneuropathy, mononeuropathies, and those caused by traumatic or surgical nerve damage 1,3.

Epidemiologic data suggest that NP affects up to 8% of the population in Europe 4–6, and current treatments include tricyclic antidepressants, serotonin/norepinephrine reuptake inhibitors, anticonvulsants, opioids, topical lidocaine, and capsaicin creams 7,8. However, these existing treatments typically provide no more than half of patients with satisfactory pain relief 7. Moreover, systemic therapies are often associated with side effects, such as nausea, dizziness, and somnolence, which are unpleasant and can contribute to the burden of the disease itself 7,8.

The capsaicin 8% patch (Qutenza™) is an alternative treatment option for NP that is designed to deliver a high dose of capsaicin topically where the pain is experienced. Capsaicin, the active component of chili peppers, is an agonist of transient receptor potential vanilloid 1 (TRPV1) channels, which play an important role in the transmission of pain signals 9. Continuous activation of TRPV1 causes nociceptor defunctionalization—accompanied by a reversible reduction in epidermal nerve fiber density—and an inhibition of pain transmission 10. This results in a prolonged, but reversible, reduction in the symptoms of peripheral neuropathic pain (PNP) 11–13.

The capsaicin 8% patch was approved in the European Union (EU) in 2009 for the treatment of PNP in nondiabetic adults either alone or in combination with other medicinal products based on an extensive clinical trial program investigating its efficacy and safety for the treatment of NP, particularly in patients with PHN or HIV-AN 12. Results from the Phase III trials demonstrated that a single 30- or 60-minute application of the capsaicin 8% patch significantly reduced pain for 3 months in patients with HIV-AN or PHN, respectively 14–16. The capsaicin 8% patch retains its efficacy upon re-treatment 17,18 and is effective both as a monotherapy and when used in conjunction with concomitant medications for NP 14–16. In the EU, the capsaicin 8% patch is not licensed for use in patients with diabetes due to lack of data from this patient population as studies are still ongoing.

In Phase III trials, the capsaicin 8% patch was generally well tolerated. The majority of adverse events were mild to moderate transient application-site reactions, such as pain and erythema 14,15. In addition, safety studies have showed that there was no neurological or sensory impairment in patients who received repeated treatments with the capsaicin 8% patch over a period of 1 year 17,19.

The capsaicin 8% patch is a relatively new treatment modality in Europe, and here the authors describe an analysis of over 60 patients who have received this treatment at the Clinic for Pain Therapy and Palliative Medicine at the Medical Centre for the region of Aachen, Germany. Patients are treated for a variety of PNP conditions at this center, including PHN, radiculopathy, polyneuropathy, FBSS, facial neuropathy (severe trigeminal neuralgia in V2), and different types of mononeuropathies including posttraumatic and surgical. The aim of this retrospective analysis was to evaluate the efficacy and tolerability of the capsaicin 8% patch in a real-life clinical setting as a treatment for a variety of PNP conditions, including a number that were not investigated during the pivotal clinical trials.

## Methods

### Patients

All patients with a diagnosis of PNP attending the clinic for treatment with the capsaicin 8% patch were included in the analysis. Patients receiving capsaicin 8% patch treatment included some with FBSS and radiculopathy whose pain was neuropathic in nature. Most patients were treated with the capsaicin 8% patch in accordance with the manufacturer's treatment guidelines. Exceptions were that two patients were treated on the face for severe trigeminal neuralgia in V2, 15 patients were treated with the capsaicin 8% patch without topical anesthetic pretreatment (please see below), and five patients were re-treated with the capsaicin 8% patch less than 90 days following their prior treatment, at Days 55, 57, 77, and 84.

Duration of illness was estimated as the difference in months between the date of PNP diagnosis (depending on recall precision, the mid-month, or mid-year date was used) and the date of first treatment with the capsaicin 8% patch. All treatments and assessments were consistent with usual standard of care.

### Procedure

The application procedure for the capsaicin patch was carried out as described previously 20. The painful area to be treated was identified using pinpricks and/or a brush and was marked on the patient's skin; the markings were used to match the size of the capsaicin 8% patch to the treatment area. As recommended in the product prescribing information 21, most patients were pretreated with the topical anesthetic cream EMLA (lidocaine 2.5%/prilocaine 2.5%; AstraZeneca GmbH, Wedel, Germany) for 60 minutes before treatment with the capsaicin 8% patch. EMLA analgesia persists for 1–2 hours following removal of the cream from the skin 22. Overall, 15 patients were treated with the capsaicin 8% patch without topical anesthetic pretreatment, either due to an allergy to the topical anesthetic or because they chose not to receive pretreatment.

Following removal of the topical anesthetic, the capsaicin 8% patch was applied for 30 minutes to the feet and for 60 minutes to all other areas of the body. To enhance adhesion, the patch was either bandaged to the treatment site using elastic bandages or pressed against the skin using sandbags. After patch removal, the area was cleaned with cleansing gel to remove any residual capsaicin, and cooling measures, such as cool packs wrapped in thin cloth placed on the treatment area, were initiated to reduce any treatment-related discomfort. The duration of cooling was variable, depending on the needs of each individual patient, and it was recommended that patients continue to use cooling measures at home, as necessary.

Treatment-related discomfort was monitored during and after the application procedure; for moderate pain, patients were offered oral analgesic medication, such as metamizol and/or cooling measures, while for more severe pain, patients were offered intravenous opioid treatment, such as oxycodone. Although not available in all countries, metamizol is an effective analgesic and is commonly used in Germany.

### Efficacy Measurements

Pain levels were assessed using the 11-point Numeric Pain Rating Scale (NPRS), asking patients the question “what is your pain now?” All patients received a baseline assessment in the clinic on the day of capsaicin 8% patch treatment to determine pretreatment pain levels. The patient's pain was assessed immediately after capsaicin 8% patch treatment, and then on Days 1 and 3, and during Weeks 1, 4, 8, and 12 following. Follow-up was carried out by telephone call or face to face clinic contact on Days 1 and 3, and Weeks 1, 4, 8, and 12.

Change in pain was assessed both in absolute terms (unit of NPRS change) and as a percentage change from baseline (prior to the first capsaicin 8% patch treatment). Pain response to the first capsaicin 8% patch treatment was calculated as the percentage change between the baseline NPRS value and the average of NPRS scores at Weeks 1, 4, and 8. As in the capsaicin 8% clinical trials 14–16,18, a responder was defined as a patient who exhibited a ≥30% decrease in NPRS score from baseline.

### Re-Treatment

Patients were re-treated with the capsaicin 8% patch when their pain significantly increased and they were in discomfort again; pain did not have to return to baseline levels for re-treatment to be undertaken.

### Prescribed Concomitant Pain Medication Use

Prescribed concomitant pain medications taken by patients were assessed throughout the follow-up period, and these medications were modified as appropriate. At the 4-week follow-up, potential changes to prescribed concomitant NP medications were discussed with the patient and initiated, as appropriate. Prescribed concomitant NP medications were recorded, and classified as opioids, anticonvulsants, or antidepressants.

### Statistical Analysis

Inferential tests appropriate to the distribution of the dependent variables were selected. Within-subject differences before and after treatment were assessed by paired-samples comparison, pain score differences from baseline to follow-up by paired sample *t*-test, and concomitant pain medication change by Wilcoxon signed ranks. Subgroup differences were analyzed with independent-samples comparisons; one-way analysis of variance was used to assess difference in percentage change in pain between PNP subtypes.

The re-treatment interval was calculated by the Kaplan–Meier method 23, and the log-rank test was used to assess between-group differences. Patients who did not receive re-treatment were censored at the time of data lock (February 7, 2011). This assumed that all censored patients were eligible for re-treatment unless specifically indicated otherwise by an intolerable adverse reaction to treatment (as judged by the clinician), a patient-indicated preference not to receive re-treatment with the capsaicin 8% patch, or a patient being lost to follow-up. In such cases, the patient was censored at 90 days.

All inferential tests were conducted using SPSS for Windows (version 18; IBM, New York, USA). The threshold significance level for all tests was set at *P* = 0.05.

## Results

### Patients

Overall, 68 patients received a total of 96 treatments with the capsaicin 8% patch at the Clinic for Pain Therapy and Palliative Medicine at the Medical Centre for the region of Aachen, Germany, between January 13, 2010 and February 7, 2011. Of these patients, six had been diagnosed with FBSS or radiculopathy, two with facial neuropathy (severe trigeminal neuralgia in V2), six with polyneuropathy, 20 with PHN, and 34 with other types of peripheral neuropathy, which included patients with posttraumatic or surgical nerve injuries and mononeuropathies (Table 1). Almost half of the patients were male, and the median duration of NP was nearly 2 years. The majority of patients were taking some form of NP medication at the time of treatment with the capsaicin 8% patch (Table 1).

**Table 1:** Baseline characteristics of patients (N = 68) treated with the capsaicin 8% patch

Characteristics	Value
Male, N (%)	29 (42.6)
Age, mean (SD), years	59.9 (13.3)
Duration of PNP, median (IQR), months	22 (8–52)
Baseline NPRS score, mean (SD)	7.5 (1.6)
PNP diagnosis, N (%)	
FBSS, radiculopathy	6 (8.8)
Neuropathy (face)	2 (2.9)
Peripheral neuropathy	34 (50.0)
Polyneuropathy	6 (8.8)
PHN	20 (29.4)
Type of prescribed concomitant PNP medication(s) at baseline, N (%)	
Opioid	34 (50.0)
Anticonvulsant	31 (45.6)
Antidepressant	28 (41.2)
Prescribed concomitant PNP medication(s) at baseline, N (%)	
0	12 (17.6)
1	23 (33.8)
2	29 (42.6)
≥3	4 (5.9)

FBSS = failed back surgery syndrome; IQR = interquartile range; NPRS = Numeric Pain Rating Scale; PHN = post-herpetic neuralgia; PNP = peripheral neuropathic pain; SD = standard deviation.

Of the 68 patients who received treatment with the capsaicin 8% patch, 22 received two treatments, five received three treatments, and one patient received four treatments. Eight-week follow-up data are available for 53 of the 68 patients, and 12-week follow-up data are available for 44 of these patients. The remaining patients had not yet reached their scheduled 8- or 12-week follow-up.

### Efficacy

#### NPRS Score

**Figure 1:**
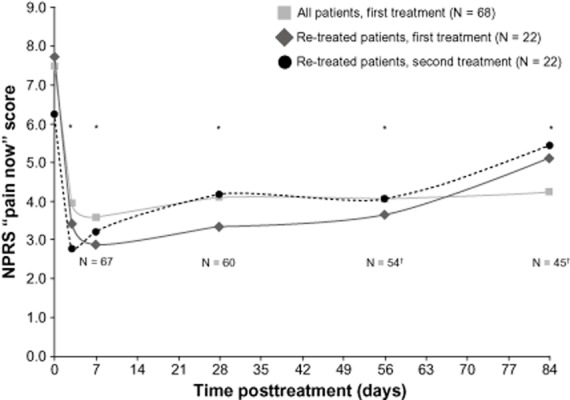
Mean NPRS score after treatment with the capsaicin 8% patch. The absolute NPRS scores on Days 0, 3, 7, 28, 56, and 84 posttreatment are shown for patients who were treated once with the capsaicin 8% patch and for patients who received a second treatment. * *P* < 0.001 for all patients, first treatment. ^†^ One patient missed the 4-week follow-up, and therefore could not be included in the responder analysis of patients who had a complete follow-up of at least 8 weeks (N = 53; however, 8-week data were available for this patient, so they were included in the data presented here. Likewise, 12-week data were available for N = 45 patients, but only N = 44 patients had a complete follow-up of 12 weeks. NPRS = Numeric Pain Rating Scale.

In all patients (N = 68), the decrease in pain after treatment with the capsaicin 8% patch occurred rapidly and was evident from 7 days posttreatment (Figure 1). At Day 7, there was a significant (*P* < 0.001) mean reduction in NPRS score compared with baseline. A significant reduction in pain was maintained up to 12 weeks posttreatment; those patients followed up at 12 weeks (N = 44) still exhibited a reduction from baseline pain score of 43.4% (95% confidence interval [CI] 31.3–55.5%; *P* < 0.001).

**Figure 2:**
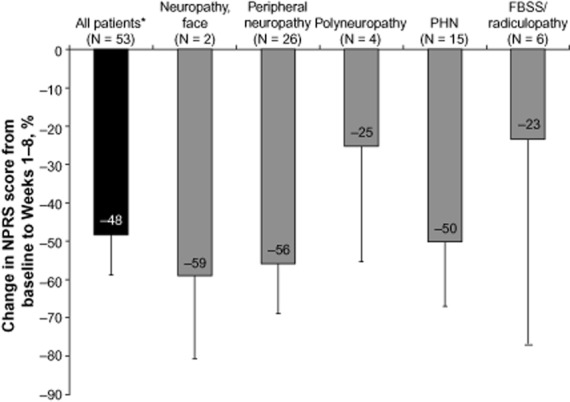
Mean (95% confidence interval) percentage reduction in NPRS score from baseline to Weeks 1–8 following treatment with the capsaicin 8% patch, in those patients with a follow-up of at least 8 weeks. * Patients who received a follow-up of at least 8 weeks. FBSS = failed back surgery syndrome; NPRS = Numeric Pain Rating Scale; PHN = post-herpetic neuralgia.

**Figure 3:**
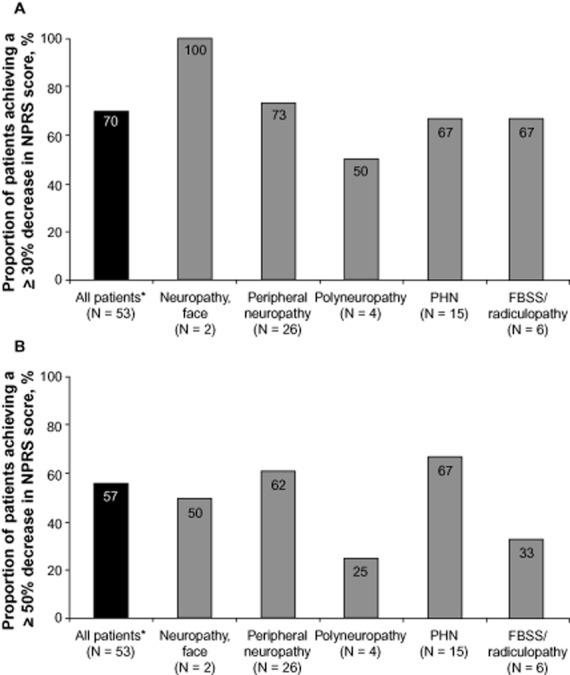
Proportion of patients who responded to treatment with the capsaicin 8% patch from baseline to Weeks 1–8, in those patients with a follow-up of at least 8 weeks. The proportion of patients who experienced (A) ≥30% decrease or (B) ≥50% decrease in NPRS score overall and by indication. * Patients who received a follow-up of at least 8 weeks. FBSS = failed back surgery syndrome; NPRS = Numeric Pain Rating Scale; PHN = post-herpetic neuralgia.

Among patients with a follow-up of at least 8 weeks (N = 53), the mean reduction was 48.4% (95% CI 38.0–53.7%; *P* < 0.001) (Figure 2). Similarly, analysis of reduction in NPRS score by NP type demonstrated that treatment with the capsaicin 8% patch caused a comparable (*P* = 0.282) decrease in pain intensity for patients with all types of NP investigated. Of the 53 patients with ≥8-week follow-up, 70% (N = 37) responded to treatment with the capsaicin 8% patch (reduction of ≥30% in NPRS score from baseline to Weeks 1–8; Figure 3A). There was a high proportion of responders for all five NP types treated with the capsaicin 8% patch, ranging from 50% in patients with polyneuropathy to 100% in patients with facial neuropathy. The majority (57%; N = 30) of these patients also showed a reduction of ≥50% in NPRS score from baseline to Weeks 1–8 (Figure 3B).

### Prescribed Concomitant Pain Medication

**Figure 4:**
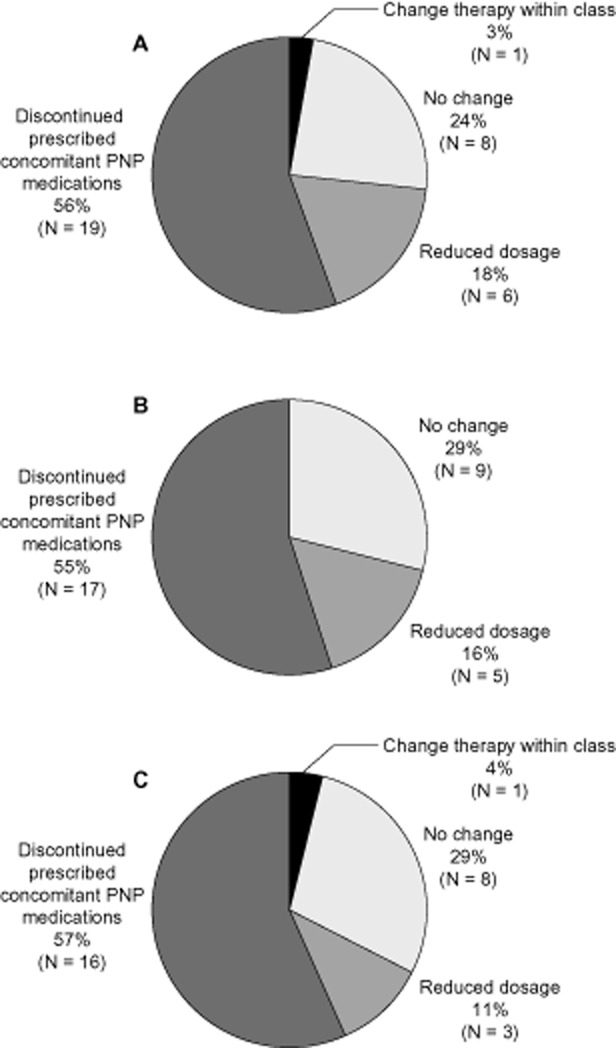
Changes in the use of prescribed concomitant peripheral neuropathic pain (PNP) medications following the first treatment with the capsaicin 8% patch. Changes in the use of (A) opioids, (B) anticonvulsants, and (C) antidepressants. Percentages add up to >100% due to rounding errors.

There was a significant (*P* < 0.001) reduction in the prescribed concomitant NP medication (opioids, anticonvulsants, or antidepressants) taken by all patients, and those classed as responders, following treatment with the capsaicin 8% patch (Table 2). At the time of capsaicin 8% patch treatment, patients took a mean of 1.37 types of medication for their NP, but following a single application of the capsaicin 8% patch there was a 54% reduction in the mean number of medications taken by each patient (Table 2). Moreover, among patients previously receiving opioids, anticonvulsants, or antidepressants, more than half discontinued this medication, and a further 18%, 16%, and 11%, respectively, reduced their daily dose (Figure 4).

**Table 2:** Prescribed concomitant PNP medication use with the capsaicin 8% patch

Prescribed PNP Medication	All Patients (N = 68)	Responders* (N = 37)
No. of prescribed PNP medication(s), mean (SE)		
Pretreatment	1.37 (0.102)	1.46 (0.148)
Posttreatment	0.63 (0.089)	0.73 (0.120)
Reduction in prescribed PNP medication use, %	54	50
*P* value	<0.001	<0.001

* Change in Numeric Pain Rating Scale score of ≥30% from baseline to Weeks 1–8.

PNP = peripheral neuropathic pain; SE = standard error.

Of 56 patients receiving ≥1 prescribed concomitant pain medication prior to treatment with the capsaicin 8% patch, 23 (41%) discontinued all other prescribed pain medications after receiving a single treatment (Table 3). Of the four patients who took all three types of prescribed pain medication before treatment with the capsaicin 8% patch, two discontinued use of all other prescribed pain medications following treatment, one patient discontinued two of their three prescribed NP medications, while the other patient discontinued one of their three prescribed NP medications. Only one patient, who was a nonresponder, increased the number of prescribed NP medications they were taking following capsaicin 8% patch treatment.

**Table 3:** Prescribed concomitant PNP medication discontinuations following treatment with the capsaicin 8% patch

No. of Prescribed Concomitant PNP Medications Being Received at Baseline	No. of Patients (%) Receiving 1, 2, or 3 Prescribed PNP Medication(s) at Baseline (N = 56)	No. of Patients (%) Discontinuing All Prescribed Concomitant PNP Medications after Capsaicin 8% Patch Treatment
1	23 (41)	13 (57)
2	29 (52)	8 (28)
3	4 (7)	2 (50)

PNP = peripheral neuropathic pain.

#### Re-Treatment

Twenty-two patients were re-treated with the capsaicin 8% patch between 8 and 21 weeks after the first treatment. Prior to re-treatment, mean pain levels in these patients remained significantly (*P* = 0.017) lower than the original mean baseline levels recorded before the first treatment (NPRS score 6.23 vs 7.72, respectively). The reduction in NPRS score observed after re-treatment with the capsaicin 8% patch was similar to that observed after the initial treatment (Figure 1). At Day 7 following re-treatment, the mean NPRS score was 3.2 compared with 2.9 on Day 7 following the first treatment in these patients. The reduction in pain was maintained at a similar level over 12 weeks following re-treatment with the capsaicin 8% patch as after the initial treatment (Figure 1).

The mean reduction in NPRS score from baseline to Weeks 1–8 was also similar following re-treatment compared with the first treatment (43% vs 52%, respectively; *P* = 0.552) in the 16 patients who were observed for 8 weeks following re-treatment. Of these patients, 63% (N = 10) exhibited a ≥30% reduction in NPRS score from baseline to Weeks 1–8.

Among the responders to first capsaicin 8% patch treatment, that is, those patients who exhibited a ≥30% reduction in NPRS score from baseline to Weeks 1–8 following their first treatment (20 of the 22 re-treated patients), the median time to re-treatment after application of the first patch was 125 days (95% CI 93–157 days), and the mean was 191 days (95% CI 151–230 days).

### Tolerability

All patients completed 100% of the intended capsaicin 8% patch application time. Only four patients required analgesia for application-related discomfort during their first capsaicin patch application, a single dose of a short-acting opioid (oral metamizol in three cases, the other intravenous oxycodone). One patient required medication with metamizol, 1.5 g, during re-treatment. All patients were provided with cooling measures to reduce treatment-related discomfort following patch removal, and patients were advised to continue to use cooling measures at home, as necessary. No other adverse events were reported.

## Discussion

This retrospective analysis demonstrated that treatment with the capsaicin 8% patch in a real-life clinical setting significantly reduced PNP for at least 12 weeks in several conditions, including patients with FBSS/radiculopathy whose pain was neuropathic in nature, facial neuropathy (severe trigeminal neuralgia in V2), peripheral neuropathy, polyneuropathy, and PHN, which affect various sites of the body and are caused by different mechanisms. There did not appear to be any difference in response to the treatment between patients from each diagnostic subtype, although this analysis was clearly not powered to show equivalence. These data, which highlight the efficacy of the capsaicin 8% patch in several NP populations, are important because the capsaicin 8% patch has a broad license, being approved for the treatment of PNP in nondiabetic adults either alone or in combination with other medicinal products for pain. While the clinical trial program demonstrated efficacy in patients with PHN and HIV-associated distal sensory polyneuropathy (HIV-DSP), this analysis clearly demonstrates the efficacy of the capsaicin 8% patch in a much wider range of NP populations.

This is the first article of which we are aware that reports an analysis of the efficacy of the capsaicin 8% patch in clinical practice. Overall, the pain reduction observed in this cohort of patients in a real-life setting was larger than that seen during the Phase III clinical trials 14–16. In clinical trials, patients with PHN reported a mean percentage change from baseline in NPRS score of −32.7% during Weeks 2–4 17 and −29.6% to −32.0% during Weeks 2–8 14,16. This is compared with a mean change in NPRS of −48.4% from baseline to Weeks 1–8 for all patients in this retrospective analysis and −50.3% for the subset of patients with PHN. Furthermore, the proportion of responders in the patients treated in the clinical setting was also greater than in the clinical trials. Overall, 69.8% of patients treated at the clinic in Aachen exhibited a ≥30% reduction in NPRS score and 66.7% of patients with PHN were responders vs 42% and 46%, respectively, of patients with PHN treated during the clinical trial program 14,16. There are a number of possible explanations for the improved response rate observed in our clinic in Aachen compared with clinical trial results. It is possible that variations in the methods used for determining pain levels and assessing pain reductions in the clinical trials vs the real-life clinical setting could contribute to this difference, or that patients perceive pain differently in a clinical setting compared with a clinical trial. However, it is more likely that the enhanced efficacy seen in our analysis is due to improved capsaicin 8% patch application techniques 20. As experience in the clinical use of the capsaicin 8% patch has accumulated, it has become clear that a number of factors may influence optimal treatment, including ensuring complete contact between patch and skin, and maximizing adhesion using bandaging.

The analgesic effect of the capsaicin 8% patch was rapid, being evident within 1 day post-application. This fast onset in patients treated in the clinic was consistent with data from the published data in PHN 14,16,24. Response was also rapid following re-treatment with the capsaicin 8% patch, with mean NPRS scores of 2.92 and 3.13 at Day 7 following re-treatment and the first treatment, respectively. As with pain reductions after the first capsaicin patch treatment, the mean reduction in NPRS score following re-treatment in the clinic setting was greater than that observed in clinical trials (43% vs 31.4% for patients with PHN and 21% for patients with HIV-DSP, respectively 17,18), as was the proportion of responders (63% vs 35% of patients with HIV-DSP 18). However, it should be noted that the clinical trial data were determined for Weeks 2–12, while the data in this retrospective analysis were determined for Weeks 1–8, which might contribute to this difference.

As previously demonstrated for patients with PHN and HIV-DSP 18,25, this retrospective analysis confirms that the efficacy of the capsaicin 8% patch is maintained following re-treatment. Moreover, here the efficacy of re-treatment in a wider NP population has been demonstrated. The analysis also demonstrated that the duration of response is greater than 90 days, as evidenced by the median time to re-treatment, which was 125 days. This duration of response is remarkably consistent to that determined for patients with HIV-DSP in the clinical trials; these patients exhibited a median time to first re-treatment of 126 days 18. This suggests a similar duration of response in our clinical practice to that observed previously in clinical trials. Also of interest is the high proportion of responders (those exhibiting a ≥30% reduction in NPRS score from baseline to Weeks 1–8) who did not require re-treatment (38%). Including these censored cases, the mean re-treatment interval in this analysis was 191 days, consistent with open-label clinical trial extension data in PHN 25, further confirming that responders to treatment with the capsaicin 8% patch experience prolonged reductions in pain.

Systemic treatments for PNP are associated with unpleasant side effects that can contribute to the burden of the disease itself 7,8. Our analysis demonstrated that treatment with the capsaicin 8% patch allowed patients to significantly reduce the mean number of prescribed concomitant PNP medications they were taking, potentially improving their health-related quality of life (QoL). Although the impact of a reduction in prescribed concomitant medications on health-related QoL has not been formally assessed in a clinical trial in this setting, an integrated analysis across four clinical trials in PHN reported that QoL was significantly improved following capsaicin 8% patch treatment 26. This is the first time that changes in prescribed concomitant medication following capsaicin 8% patch treatment have been evaluated because the clinical trial protocols fixed PNP medications at screening in order to measure the true analgesic effect of the capsaicin 8% patch.

Pharmacy costs are significant cost drivers of PNP treatment. A retrospective study using the MarketScan® database reported that among PHN patients in the United States, excess total costs for these patients resulted from higher costs for outpatient/professional services (54%) and pharmacy (45%) 27. Therefore, the reduction in prescribed concomitant medication use following capsaicin 8% patch treatment should offset some of the total treatment cost for the patient, although further analyses are required to confirm this.

The application of the capsaicin 8% patch was generally well tolerated in this real-life setting, with a low proportion of patients (6%) requiring medication for application-related discomfort during the first capsaicin 8% patch application. Medication was not required by any patients during re-treatments, and there were no early discontinuations. Interestingly, the proportion of patients who required medication for application-related discomfort during this study was lower than in the clinical trials (15–44%; 14–16). Reasons for this are currently unclear. One explanation may be that cooling of the application site immediately following removal of the capsaicin 8% patch, which we have found to be very effective at reducing discomfort, is routine in our clinical practice. However, it should be noted that cooling during patch application is currently not recommended, as the potential impact of low temperature on the efficacy of the capsaicin patch has not yet been properly assessed. Another explanation could be that during the clinical trials, centers were provided with medication to manage application-related discomfort and were proactively advised to administer to patients. It may also be pertinent that the majority of our patients described the treatment-associated discomfort as a burning sensation rather than a pain, and found it quite manageable.

As a retrospective analysis, this study is limited by a lack of randomization and placebo control, and only represents experience from a single clinic. Consequently, additional analysis of patient responses to the capsaicin 8% patch in other real-life clinical settings will be required to validate the data presented here. Additional studies across a wider variety of PNP conditions are also warranted, and patient benefits relating to the reduction in the use of concomitant medications should be explored further. Nevertheless, the data presented here support the efficacy of the capsaicin 8% patch to treat NP and suggest that response rates may even be higher when used in real-life clinical practice than they were during clinical trials. Furthermore, the data show that the capsaicin 8% patch can provide rapid and sustained reductions in pain levels in patients with a variety of PNP conditions, together with a significant reduction in the requirement for prescribed concomitant pain medications. These data indicate that the capsaicin 8% patch will be a very useful addition to the treatment options available for NP.
